# Utility of High Fluorescent Cell Parameter on Automated Hematology Analyzer in Screening for Dengue Infection and Predicting Platelet Recovery: Objective and Cost-Effective Solution

**DOI:** 10.7759/cureus.79048

**Published:** 2025-02-15

**Authors:** Richa Juneja, Shalaka Khade, Kalaiselvi Selvaraj, Tanul Jain, Rasika Gadkari

**Affiliations:** 1 Pathology, All India Institute of Medical Sciences, Nagpur, Nagpur, IND; 2 Community Medicine, All India Institute of Medical Sciences, Nagpur, Nagpur, IND; 3 Community Medicine, All India Institute of Medical Sciences, Madurai, Madurai, IND

**Keywords:** activated lymphocytes, dengue, hematology analyzer, high fluorescent cells, platelet

## Abstract

Background and objective

Dengue is a common febrile illness in tropical countries; serological detection of non-structural protein-1 (NS1) antigen or IgM is the gold standard for diagnosis. Thrombocytopenia, leukopenia, eosinopenia, and hem concentration are common hematological manifestations in dengue, and activated lymphocytes are seen in these patients. We intend to analyze the utility of a parameter called a high fluorescent cell (HFC) on Mindray BC-6000 for suspecting dengue infection in patients with febrile illness. Thrombocytopenia, when present, is taken as a warning sign and creates serious concerns for the treating physician. We studied HFC as a cost-effective tool to predict platelet recovery.

Methods

This is a facility-based single-gate cross-sectional comparative diagnostic accuracy study. Patients presenting with febrile illness and undergoing dengue serology testing and complete blood count (CBC) test with HFC enumeration on the same day were analyzed. Dengue-positive patients with thrombocytopenia were serially monitored for both platelet count and HFC, along with other platelet parameters.

Results

A total of 515 febrile patients were included. The median age of the patients was 18 (11-31) years, and 281 (54.56%) patients were males. Overall, the lab positivity rate for dengue was 33% (170 patients out of 515). The HFC in dengue-positive patients ranged from 0 to 20.1%. The sensitivity and specificity of absolute HFC count at a cut-off of 0.02 were 74% and 32%, respectively; similarly, the sensitivity and specificity of HFC percentage at a cut-off of 0.2 were 92% and 25%, respectively. Eighty-nine (out of 170) dengue-positive patients had thrombocytopenia. In 15 cases, multiple serial HFC and platelet counts were available. The falling trend in HFC was followed by platelet recovery within 24 hours in 13 (86.6%) patients.

Conclusion

HFC serves as a sensitive but not specific marker for dengue infection in cases with febrile illness. The role of HFC in predicting platelet recovery in dengue cases should be further explored.

## Introduction

Dengue is one of the common arthropod-borne febrile illnesses in tropical countries. Its severity varies from mild illness without any warning signs to severe forms like dengue hemorrhagic fever and dengue shock syndrome [1]. Serological detection of non-structural protein-1 (NS1) antigen or IgM by card test or enzyme-linked immunosorbent assay (ELISA) is the gold standard for establishing the diagnosis [2]. Thrombocytopenia, leukopenia, and lymphocytosis are common hematological manifestations in dengue, and activated lymphocytes are seen in these patients [3-5]. Thrombocytopenia is detected in 66 to 88% of patients [3,6,7], and a rapid decline in platelet count can cause significant concern. Furthermore, if the patient receives a platelet transfusion, it increases the risk of alloimmunization and causes economic loss when platelet recovery is just impending.

Presently, several platelet parameters like platelet large cell ratio (PLCR), mean platelet volume (MPV), platelet distribution width (PDW), and, most importantly, immature platelet fraction (IPF) available on automated hematology analyzers are studied as potential predictors of platelet recovery. All these depend on detecting large platelets by counters [8]. However, IPF needs special fluorescent dye, which causes additional costs to the patient.

For all the patients presenting with fever, a complete blood count is always performed. Recent literature shows a parameter called high fluorescent lymphocyte represents activated lymphocytes on the Sysmex (Sysmex Co., Kobe, Japan) complete blood count (CBC) analyzer and has good sensitivity and specificity in detecting dengue infection [9].

We aimed to study the utility of a similar parameter called a high fluorescent cell (HFC) on Mindray BC-6000 (Shenzhen Mindray Bio-Medical Electronics Co., Ltd., Shenzhen, China) for suspecting dengue infection in patients with febrile illness and to study the utility of HFC in predicting platelet recovery in dengue patients as fall in activated lymphocytes is usually followed by platelet count recovery.

## Materials and methods

The study was approved by the institutional ethics committee at the tertiary care hospital in Central India. This was a facility-based single-gate cross-sectional comparative diagnostic accuracy and retrospective study.

Febrile patients with temperatures more than 100.4°F (38°C) presenting to the institute from 1 July 2021 to 31 August 2021 were included in the study. As per the institute protocol, all patients presented with febrile illness underwent a complete blood count, including peripheral smear for malaria, reverse transcription polymerase chain reaction (RT PCR) for COVID-19 based on suspect criteria and NS1 dengue and IgM dengue. Patients admitted to the hospital with dengue infection and thrombocytopenia were typically severe or complicated cases. This included individuals experiencing significant platelet drop, bleeding tendencies, organ involvement, or other warning signs like severe abdominal pain, persistent vomiting, fluid accumulation, or difficulty breathing. These patients required close monitoring and supportive care. Among these inpatients, periodic platelet counts were assessed to predict the prognosis and plan for the discharge. On average, each admitted thrombocytopenic patient undergoes 2-3 serial platelet assessment during their stay in the hospital.

For sensitivity and specificity of HFC for dengue infection, all the patients with febrile illness undergoing dengue serology and at least one CBC test with HFC enumeration were included regardless of their platelet count level. All dengue-positive (NS1 or IgM) thrombocytopenic patients (platelets less than 1.5 lac/cu mm) with serial CBC/HFC enumeration were included to assess HFC as a predictor of platelet recovery. Patients with a history of platelet transfusions in the past 15 days were excluded from the study, as this could affect the CBC results. Also, only IgG-positive dengue cases were excluded, as they might represent past infection and not active cases.

Assuming the area under the curve for HFC 0.898, as reported by Jayaram et al. [9], and 10% relative precision, 5% alpha error, and 20% prevalence of disease, the minimum number of dengue cases required was 108 and thus a total number of febrile patients estimated to be 500.

Detection of dengue antigen by serology is the gold standard for dengue diagnosis. Rapid kits were used for dengue serology, which detects both dengue virus NS1 antigen and differential IgM/IgG antibodies to dengue virus in human whole blood, serum, or plasma. The principle lies in the formation of an antigen-antibody complex. When the specimen is added to the well, anti-dengue IgG, IgM, or NS1 reacts with recombinant dengue virus envelope proteins-colloidal gold conjugates, which form the antigen-antibody complex. This complex develops a colored line when it migrates along the device by capillary action.

**Figure 1:**
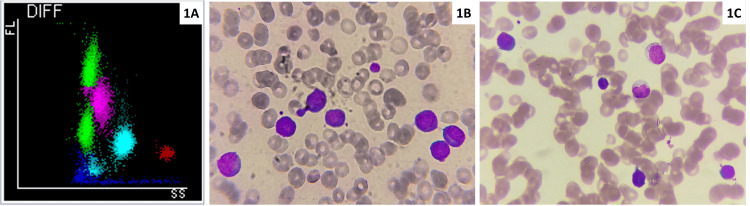
Scattergram (A) and peripheral smear (B, C) A: CBC scattergram showing an 8-like pattern of lymphocytes with activated lymphocytes forming a cluster with higher fluorescence (green - lymphocytes, pink - monocytes, light blue - neutrophils, red - eosinophils). B and C: Peripheral smear showing activated lymphocytes (Leishman stain, ×1000) being counted as HFC by the analyzer. CBC - complete blood count; FL- fluorescent signal; SS - side scatter; HFC - high fluorescent cell

CBC was performed on ethylenediaminetetraacetic acid (EDTA) anticoagulated blood sample using the fully automated hematology analyzer Mindray BC-6000. This analyzer works on the principle of SF-Cube technology for white blood cell (WBC) differential count and flagging of abnormal cells. After reaction with proprietary reagents, the targeted blood cells undergo 3D analysis using information from the scatter of laser light at two angles: one at the forward direction, called forward scatter, and the other at 90°, called side scatter and fluorescence signals. The 3D scattergram builds the power to better identify and differentiate blood cell populations, especially to reveal abnormal cell populations undetected by other techniques. HFC parameters represent high fluorescent cell populations, such as blasts and atypical lymphocytes as indicated in the images below. HFC percentage and absolute count are routinely obtained as research parameters in CBC and do not incur any additional expenditure to the patient (Figure 1). The normal range for HFC was determined using 50 normal healthy persons. HFC values from CBC done on the day of dengue testing were taken to establish the utility of HFC in predicting dengue infection. Serial HFC was recorded for dengue-positive thrombocytopenic patients wherever available and compared with platelet count till recovery.

The objectives of the study were to identify the specific cut-off for HFC to delineate the presence or absence of dengue infection; to estimate the sensitivity, specificity, and positive and negative predictive value of HFC for dengue infection among patients presenting with febrile illness in hospital; and to study the utility of HFC to predict the time taken for platelet recovery among dengue patients with thrombocytopenia.

Diagnostic accuracy of HFC against the conventional NS1 or IgM dengue is reported as sensitivity, specificity, positive predictive value, and negative predictive values with 95% CI. In positivity for either one of NS1 or IgM, dengue is considered a reference test (gold standard) positive. Positivity at a specific cut-off based on HFC is considered an index test positive. The specific cut-off to determine the sensitivity and specificity of HFC is estimated using the receiver operating characteristics curve. The cut-off which has the maximum Youden's criteria is chosen as a cut-off to asses the index test further.

## Results

The total number of patients included in the study was 515. Of these, 281 were male (54.56%), and 234 (45.44%) were female. All the patients underwent dengue serology. Out of the total 515 patients, 140 patients (27.18%) were positive for only dengue NS1 antigen, 10 (1.94%) were positive for only IgM, 19 patients were positive for NS1 and IgM (3.68%), and one (0.19%) was positive for IgG and IgM. Overall, lab positivity for dengue was 170 (33.01%). One patient tested only for dengue IgG and was excluded from the study.

The range of normal HFC values on Mindray BC-6000 was determined from 50 normal healthy controls (blood donors). HFC absolute value (HFC#) was determined as 0-0.01, and HFC percentage (HFC%) was 0-0.11. Other lab details like WBC count, absolute values of WBC, hemoglobin, hematocrit, and platelets were also evaluated for dengue-positive patients (Table 1).

**Table 1:** Laboratory details of dengue positive patients (n=170)

Parameter		Median	Interquartile range
White blood cells		4.13	2.99-5.65
Absolute neutrophil count		2.14	1.37-3.61
Absolute lymphocyte count		1.04	0.67-1.58
Absolute monocyte count		0.36	0.25-0.55
Absolute eosinophil count		0.01	0-0.03
Haemoglobin	Male	11.5	10.3-12.6
	Female	11.5	10.05-12.95
Haematocrit		37.9	34.7-40.9
Platelet		189	122-255

**Figure 2:**
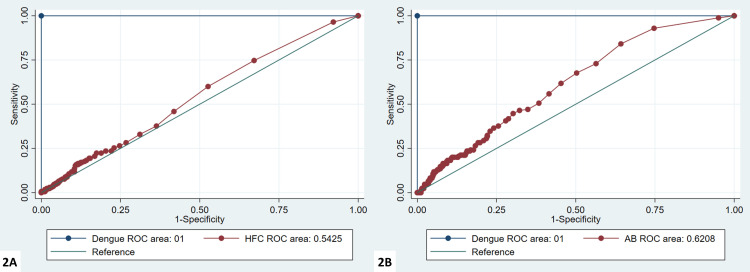
ROC curves for HFC% (A) and HFC# (B) ROC - receiver operating characteristic; HFC% - high fluorescent cell percentage; HFC# - high fluorescent cell absolute value

The HFC% in dengue-positive patients ranged from 0 to 20.1% (median 1.35). The sensitivity and specificity of HFC# and HFC % to predict dengue at different cut-offs with 95% CI were determined (Table 2). It was found that the sensitivity and specificity of HFC# at the cut-off of 0.02 was 74% and 32%, respectively; similarly, the sensitivity and specificity of HFC% at the cut-off of 0.2 was 92% and 25%, respectively. Figure 2 shows receiver operating characteristic (ROC) curve analysis of HFC% and HFC #.

**Table 2:** Sensitivity and specificity of HFC# and HFC% to predict dengue at different cut-off with 95% confidence interval (n=170) HFC% - high fluorescent cell percentage; HFC# - high fluorescent cell absolute value

Parameter		Sensitivity	Specificity	Positive predictive value	Negative predictive value
HFC#	0.02	0.74	0.32	0.35	0.72
	0.03	0.6	0.47	0.36	0.70
HFC%	0.2%	0.92	0.25	0.38	0.87
	0.3%	0.84	0.35	0.39	0.82

**Figure 3:**
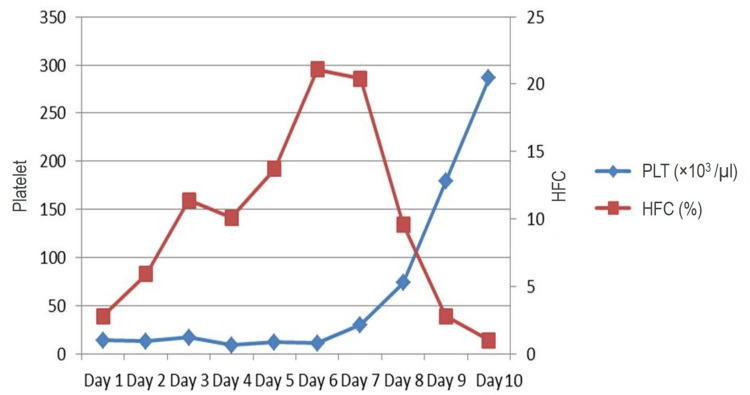
The sequential analysis of HFC and platelet count in one of the patients showed the fall in HFC preceding rise in platelet count PLT - platelet; HFC - high fluorescent cell

Out of all dengue-positive cases, 89 patients had thrombocytopenia. Sequential platelet and HFC data were available for 15 cases. In these 15 cases, platelet count at baseline varied from 5 to 109x10^3^/cu mm, and HFC% was from 0.1 to 16.3%. The falling trend in HFC was followed by platelet recovery within 24 hours in 13 (out of 15; 86.6%) patients. The peak value of HFC predicted platelet recovery in 24 hours in 10 (out of 15, 66.6%) patients and within 48 hours in three (out of 15, 20%) patients. In two patients, rising platelet count preceded a fall in HFC and peak values. In these two patients, rising HFC predicted platelet recovery in 24 hours and 72 hours, respectively. Figure 3 shows an example of one such patient where 10 sequential values of platelet and HFC were available, and peak HFC predicted a rise in platelet subsequently.

## Discussion

Dengue is characterized by arthropod-born fever, and a subset of patients develop serious complications. Thrombocytopenia is one such complication in dengue patients. It can be really severe, creating panic in both caregivers and the patient's family with an urge to give platelet transfusion. The actual risk of clinically significant bleeding is 3.4 to 7.4% in dengue [10]. Hence, early diagnosis of dengue and parameters predicting impending platelet recovery is very much needed. Another important morphological finding on peripheral smears of dengue patients is virocytes or activated lymphocytes. Dengue virus is reported to mainly affect circulating B lymphocytes. There is aberrant immune activation along with the production of cytokine IL-6, which causes abnormal plasma cell maturation and production of atypical lymphocytes [11]. Shetty et al. demonstrated that peripheral smears in dengue patients showed such activated lymphocytes with a sensitivity of 100% [11].

Activated lymphocytes can be enumerated on the hematology analyzer as high fluorescent cells on Mindray BC-6000 and high fluorescent lymphocyte cells on the Sysmex platform. This value is obtained without any additional reagent or cost during a complete blood count investigation. Activated lymphocytes are associated with increased nuclei acid along with peculiar scatter features, which are detected by an increase in fluorescent signal intensities [1]. These activated lymphocytes are postulated to be the immune reaction to dengue virus and so are found to be increased in dengue infection.

Studies have been conducted on the Sysmex platform previously to evaluate the diagnostic accuracy of these atypical lymphocytes for dengue infection [9,12-14]. A study conducted by Jayaram et al. on the Sysmex platform noted a sensitivity of 82.8% [9], while we found a sensitivity of 92% at the HFC% cut-off of 0.2%. Another study done by Raharjo et al. noted an HFC% in dengue patients in the range of 2.0 to 32.3% [13], whereas we noted this to be 0-20.1%. This difference in sensitivity across studies can be attributed to the difference in the day of checking HFC and the cut-off used. Activated lymphocytes (AL) usually rise after the subsidence of fever or before the onset of shock in dengue patients. Hence, the sensitivity of HFC for dengue diagnosis will be higher if evaluated later in the course of the disease. As we wanted to compare the accuracy with serology, we took HFC values from CBC done on the same day of serology.

The aberrant immune response in dengue infection leads to the production of autoantibodies against platelets, which leads to platelet destruction. These are IgM autoantibodies that cause lysis of platelets via complement activation. Also, it has been postulated that dengue virus infects bone marrow, leading to decreased platelet synthesis. All these mechanisms can lead to thrombocytopenia [11,15]. In the present study, amongst the dengue-positive patients, a total of 89 patients had thrombocytopenia, with 22 of them having platelet counts less than 50,000/cu mm. Lab markers are needed to predict impending platelet recovery in this group of patients to avoid transfusion.

Immature platelet fraction (IPF) on cell counters measures the reticulated platelets in peripheral blood. These reticulated platelets are distinguished from the mature platelets due to their RNA content. As the platelet production increases, the level of IPF increases. Thus, this is the indicator of the thrombopoiesis rate and can act as a biomarker to predict platelet recovery. IPF can be counted on cell counters with upgraded software and additional reagents, thus creating an additional cost [8,16].

We studied the sequential CBC of dengue-positive patients to evaluate the relation between HFC value and platelet count in 15 such patients. The falling trend in HFC was followed by platelet recovery within 24 hours in 13 (out of 15, 86.6%) patients. Similar to the study by Jayaram et al. [9], we found HFC as a promising marker to predict platelet recovery in dengue cases.

The primary limitation of this study was its retrospective design, which did not account for correlation with the day of illness at the time of HFC enumeration. Additionally, the sample size for the severe illness groups was relatively small, which may have impacted the generalizability of the findings. Future studies are warranted to validate the use of HFC further as a reliable marker for platelet recovery. To provide a more robust and comprehensive evaluation, these should include a head-to-head comparison with other established markers, such as the immature platelet fraction (IPF).

## Conclusions

In conclusion, we propose that HFC serves as a sensitive, though not specific, marker for triaging dengue infection in cases presenting with febrile illness. Its utility could be significantly increased during sudden surges of dengue cases in endemic areas. HFC offers a cost-effective means to predict platelet recovery, especially in resource-limited settings. However, further studies are necessary to establish a correlation between HFC and the timing of presentation as well as the day of sample collection to fully validate its potential as a routine screening parameter.
